# Measuring real-time synchronization in both spike trains and continuous time series

**DOI:** 10.1186/1471-2202-12-S1-P3

**Published:** 2011-07-18

**Authors:** Thomas Kreuz, Daniel Chicharro, Ralph GAndrzejak

**Affiliations:** 1 Institute for Complex Systems, CNR, Sesto Fiorentino, Italy; 2 Department of Information and Communication Technologies, Universitat Pompeu Fabra, Barcelona, Spain

## 

A wide variety of approaches to quantify the dissimilarity between two spike trains has been proposed. The Victor-Purpura metric [1] evaluates the cost needed to transform one spike train into the other using only certain elementary steps. Another metric proposed by van Rossum [2] measures the Euclidean distance between the two spike trains after convolution of the spikes with an exponential function. These methods, like many others, involve one parameter that sets the time-scale. In contrast, two more recent approaches, the ISI- and the SPIKE-distance, are parameter free and time-scale adaptive [3-5]. While the ISI-distance relies on the relative length of interspike intervals, the SPIKE-distance is sensitive to spike coincidences. Both measures are easy to visualize in a time-resolved manner and can be applied to more than two spike trains, either as averages over pairwise distances or as truly multivariate measures [6].

The SPIKE-distance is calculated from instantaneous values of spike train dissimilarity for which at each time moment not only the last previous spike but also the first following spike is taken into account. This non-causal dependence on ‘future’ spiking does not allow for a real-time calculation. Furthermore, the SPIKE-distance is designed to be applied to sequences of discrete events and does not foresee an application to continuous data (such as the electroencephalogram, EEG).

Here we address both issues and present two extensions of the SPIKE-distance. In the first extension the original method is modified such that instantaneous value of dissimilarity for two or more spike trains can be calculated in real-time. In the second extension the SPIKE-distance is transformed into a measure of dissimilarity which can be applied to two or more channels of continuous data. Both variants can also be combined such that the instantaneous synchronization among continuous time series is monitored in real-time.
